# Improvement of 2-phenylethanol production in *Saccharomyces cerevisiae* by evolutionary and rational metabolic engineering

**DOI:** 10.1371/journal.pone.0258180

**Published:** 2021-10-19

**Authors:** Linghuan Zhu, Sha Xu, Youran Li, Guiyang Shi

**Affiliations:** 1 College of Food Science and Biology, Hebei University of Science and Technology, Shijiazhuang, Hebei, China; 2 Key Laboratory of Industrial Biotechnology, Ministry of Education, School of Biotechnology, Jiangnan University, Wuxi, Jiangsu, China; 3 National Engineering Laboratory for Cereal Fermentation Technology, the Key Laboratory of Industrial Biotechnology of Ministry of Education, Jiangnan University, Wuxi, China; CNR, ITALY

## Abstract

2-Phenylethanol (2-PE) is a valuable aromatic compound with favorable flavors and good properties, resulting in its widespread application in the cosmetic, food and medical industries. In this study, a mutant strain, AD032, was first obtained by adaptive evolution under 2-PE stress. Then, a fusion protein from the Ehrlich pathway, composed of *tyrB* from *Escherichia coli*, *kdcA* from *Lactococcus lactis* and *ADH2* from *Saccharomyces cerevisiae*, was constructed and expressed. As a result, 3.14 g/L 2-PE was achieved using L-phenylalanine as a precursor. To further increase 2-PE production, L-glutamate oxidase from *Streptomyces* overexpression was applied for the first time in our research to improve the supply of α-ketoglutarate in the transamination of 2-PE synthesis. Furthermore, we found that the disruption of the pyruvate decarboxylase encoding gene *PDC5* caused an increase in 2-PE production, which has not yet been reported. Finally, assembly of the efficient metabolic modules and process optimization resulted in the strain RM27, which reached 4.02 g/L 2-PE production from 6.7 g/L L-phenylalanine without in situ product recovery. The strain RM27 produced 2-PE (0.8 mol/mol) with L-phenylalanine as a precursor, which was considerably high, and displayed manufacturing potential regarding food safety and process simplification aspects. This study suggests that innovative strategies regarding metabolic modularization provide improved prospects for 2-PE production in food exploitation.

## 1. Introduction

2-Phenylethanol (β-phenylethanol, 2-PE) is an aromatic alcohol with economic and research value that has comprehensive applications in edible essence or wine due to its favorable rose-like fragrance and stable properties [1,2]. Comprehensive applications cause considerable market demand to increase by 10%-15% every year, but 2-PE output is still in short supply. For 2-PE production, chemical synthesis and extraction processes are mainly used for industrial and high-quality products, respectively. However, traditional chemical processes for 2-PE production use benzene or methylbenzene as raw materials, which are carcinogenic. Furthermore, the purification steps when using organic reagents during 2-PE production are environmentally unfriendly, with production quality limited by unexpected by-products [3]. Extraction of 2-PE from flowers can obtain a quality product; however, it has low efficiency and high cost. Compared with these processes, biological methods have the merit of being inexpensive, having mild reaction conditions, being environmentally friendly and having a short ferment cycle. The fermentation products are not only equal to natural extraction products but also show equal economic potential with chemical products without environmental pollution risks. Thus, developing an eco-friendly and efficient platform for 2-PE production has recently become a research focus. The biosynthesis of 2-PE has already been reported in various microorganisms [4–7]. Among these, *Saccharomyces cerevisiae*, which is “generally regarded as safe”, has considerable potential for the application of 2-PE in the food industry. Moreover, *S*. *cerevisiae* has its own efficient 2-PE synthetic pathway and superior stress tolerance as reported in previous studies [8,9], making it promising for the production of high titers of 2-PE.

In *S*. *cerevisiae*, 2-PE is mainly synthesized by the Ehrlich pathway [10] from L-phenylalanine as a substrate in an enzymatic transformation process that includes the following three steps: 1) transamination of L-phenylalanine, 2) decarboxylation of phenylpyruvate, and 3) reduction of phenylacetaldehyde to 2-PE. The transamination reaction from L-phenylalanine to phenylpyruvate requires α-ketoglutarate as the amino receptor, forming L-glutamate. Phenylalanine transferase, classified as types I and II, which are encoded by *ARO8* and *ARO9*, respectively, is a key reversible enzyme for the conversion [11,12]. However, the two native transferases have different properties and influence each other in some cases. The main activity of transferase is contributed by Aro8p, whereas Aro9p catalyzes this reaction in the *aro8*Δ strain. Moreover, transcription of the *ARO9* gene is induced when aromatic amino acids exist in the medium, which differs from the low transcription level in ammonia-grown conditions [13,14]. Therefore, further research is required regarding the improved catalytic direction and efficiency of the transaminases [14,15]. Then, the decarboxylation between two unstable metabolites, phenylpyruvate and phenylacetaldehyde, is catalyzed by phenylpyruvate decarboxylase. Among the five genes (*ARO10*, *THI3*, *PDC1*, *PDC5* and *PDC6*) encoding thiamine diphosphate-dependent decarboxylases, *ARO10* was reported to be sufficient to encode phenylpyruvate decarboxylase activity in the absence of the other genes, while the transcript level of *ARO10* increased 30-fold when phenylalanine replaced ammonia as the sole nitrogen source [16,17]. It should be noted that the catalytic efficiency, regulation and substrate specificities of phenylpyruvate decarboxylases in *S*. *cerevisiae* were more complicated due to the diversity of the five decarboxylases. Afterwards, phenylacetaldehyde was catalyzed to generate phenylacetate by oxidation or 2-PE by reduction, depending on the culture conditions [12].The transamination and decarboxylation processes in Ehrlich pathway have attracted more attention and studies than oxidation or reduction.

**Fig 1:**
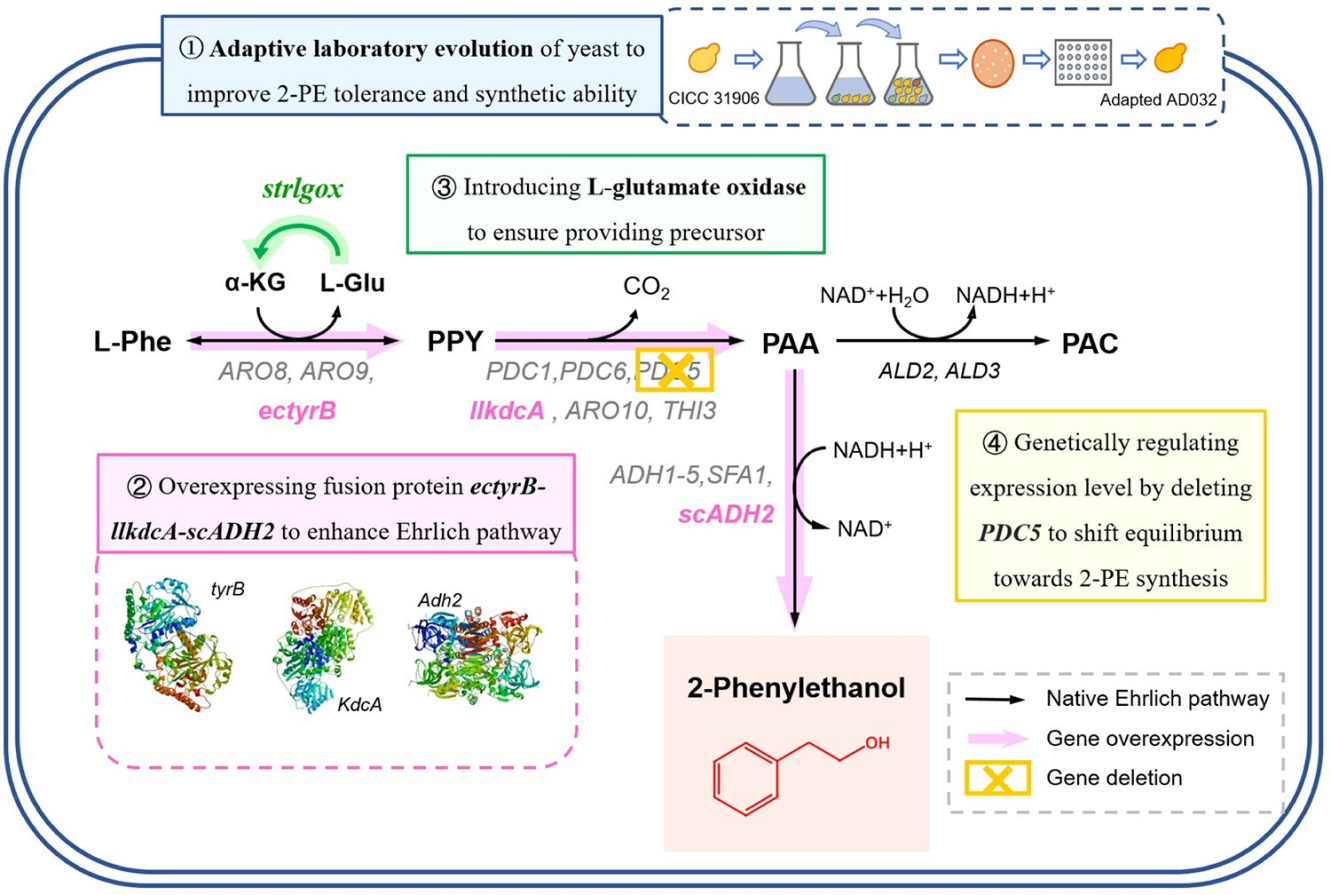
A holistic view of the engineering route in *S*. *cerevisiae* to effective 2-PE producer through Ehrlich pathway. Four categories of methods for manipulating the flux from L-phenylalanine to 2-phenylethanol were grouped and shown respectively. Metabolite abbreviations: L-Phe, L-phenylalanine; PPY, phenylpyruvate; PAA, phenylacetaldehyde; PAC, phenylacetate; 2-PE, 2-phenylethanol. Genes and enzymes: *ARO8*, *ARO9*, *tyrB*, aromatic aminotransferase; *PDC1*, *PDC5*, *PDC6*, pyruvate decarboxylase; *ARO10*, *THI3*, *kdcA*, phenylpyruvate decarboxylase; *ALD2*, *ALD3*, acetaldehyde dehydrogenase; *ADH1*, *ADH2*, *ADH3*, *ADH4*, *ADH5*, *SFA1*, alcohol dehydrogenase; *lgox*, L-glutamate oxidase.

*S*. *cerevisiae*, as a major producer of 2-PE using biotechnology, has an efficient and stable synthetic pathway that shows favorable economic potential for the industrial-scale production of 2-PE. Therefore, conventional strategies have been widely used to improve 2-PE production, including fermentation condition optimization [18,19], traditional breeding and mutagenesis [20] and in situ production removal (ISPR) [21,22], which have achieved considerable progress. Recently, based on the illumination of the biosynthesis route and regulatory mechanism of 2-PE, various metabolically engineered yeast strains have been constructed. Generally, overexpressing phenylalanine transaminase, phenylpyruvate decarboxylase and alcohol dehydrogenase in the Ehrlich pathway significantly enhanced 2-PE yield [10]. Furthermore, modifications to regulatory transcription factors, such as Aro80p and Gat1p, were applied to improve 2-PE production in *S*. *cerevisiae* as well [11,23,24]. Additionally, promoters were designed to establish a metabolic module via combinations of Ehrlich pathway components [25,26]. However, cytotoxicity has limited the high-yield production of 2-PE. Recently, to maximize the conversion of the substrate L-phenylalanine, the ISPR technique has been widely used, including resin adsorption [21], extraction [22] and membrane separation [27]. However, in view of the application of 2-PE in the food industry, the quality and safety of production are demanding. The introduction of impurities will increase the difficulty of extraction and reduce the product quality, limiting the application of 2-PE in the food industry. Considering of this, our aim is to produce 2-PE via a food safety strategy and process simplified way in *S*. *cerevisiae* as shown in Fig 1.

## 2 Materials and methods

### 2.1 Information on strains and reagents

*Escherichia coli* JM109 was used for the construction and preparation of all plasmids. The host strain was the *S*. *cerevisiae* industrial strain CICC31906, which was used for fruit wine brewing. All strains and plasmids used in this study are listed in Table 1.

**Table 1:** Strains used in this study.

Strains	Genotype	Sources
JM109	*Escherichia coli* JM109, recA1 supE44 endA1 hsdR17 gyrA96 relA1 thi (Lac-proAB) F’ (traD36 proAB+ lacI^q^ lacZM15)	Stratagene
WT-A	*Saccharomyces cerevisiae* CICC31906-α, haploid	CICC
JM00	WT-A, pYX212	This study
AD032	*S*. *cerevisiae* CICC31906-α, adaptive evolutionary strain	This study
RM08	WT-A, *klURA3*, *G418*^R^, *P*_*TPI*_*-scARO8*-*T*_*TT*_	This study
RM09	WT-A, *klURA3*, *G418*^R^, *P*_*TPI*_*-scARO9-T*_*TT*_	This study
RM10	WT-A, *klURA3*, *G418*^R^, *P*_*TPI*_*-scARO10-T*_*TT*_	This study
RM11	WT-A, *klURA3*, *G418*^R^, *P*_*TPI*_*-ectyrB-T*_*TT*_	This study
RM12	WT-A, *klURA3*, *G418*^R^, *P*_*TPI*_*-llkdcA*-*T*_*TT*_	This study
RM13	WT-A, *klURA3*, *G418*^R^, *P*_*TPI*_*-scADH2*-*T*_*TT*_	This study
RM14	WT-A, *klURA3*, *G418*^R^, *P*_*TPI*_*-scARO8-llkdcA*-*T*_*TT*_	This study
RM15	WT-A, *klURA3*, *G418*^R^, *P*_*TPI*_*-scARO9-llkdcA*-*T*_*TT*_	This study
RM16	WT-A, *klURA3*, *G418*^R^, *P*_*TPI*_*-ectyrB-llkdcA*-*T*_*TT*_	This study
RM17	WT-A, *klURA3*, *G418*^R^, *P*_*TPI*_*-ectyrB-llkdcA-scADH2*-*T*_*TT*_	This study
RM812	WT-A, *klURA3*, *G418*^R^, *P*_*TPI*_*-scARO8-scARO10-scADH2*-*T*_*TT*_	This study
RM18	WT-A, *klURA3*, *G418*^R^, *P*_*TPI*_*-strlgox*-*T*_*TT*_	This study
RM19	WT-A, *klURA3*, *G418*^R^, *P*_*TPI*_*-kitlgox*-*T*_*TT*_	This study
RM20	WT-A, *klURA3*, *G418*^R^, *P*_*TPI*_*-ectyrB-llkdcA-scADH2*-*T*_*TT*_, *P*_*TPI*_*-strlgox*-*T*_*TT*_	This study
RM21	WT-A, *pdc1*△	This study
RM22	WT-A, *pdc5*△	This study
RM23	WT-A, *pdc6*△	This study
RM24	WT-A, *aro10*△	This study
RM25	WT-A, *thi3*△	This study
RM26	WT-A, *pdc5*△, *klURA3*, *G418*^R^, *P*_*TPI*_*-ectyrB-llkdcA-scADH2*-*T*_*TT*_, *P*_*TPI*_*-strlgox*-*T*_*TT*_	This study
RM27	AD032, *pdc5*△, *klURA3*, *G418*^R^, *P*_*TPI*_*-ectyrB-llkdcA-scADH2*-*T*_*TT*_, *P*_*TPI*_*-strlgox*-*T*_*TT*_	This study

^a^ The *Saccharomyces cerevisiae* strain CICC31906 (wild type) was a fruit wine brewing strain, which was purchased from China Center of Industrial Cultural Collection.

High-fidelity Phusion DNA polymerase, restriction enzymes and *Taq*/*pfu* Master Mix were purchased from TaKaRa Bio (Takara, Shiga, Japan). Plasmid miniprep and DNA gel purification kits were purchased from Axygen Co. (Hangzhou, Zhejiang, China). All oligonucleotides (shown in S1–S4 Tables) were synthesized by Genewiz Co. (Suzhou, Jiangsu, China) All chemicals, including analytical standards, were purchased from Sigma-Aldrich (St. Louis, MO, USA) unless stated otherwise.

### 2.2 Adaptive laboratory evolution of *S*. *cerevisiae*

Yeast extract peptone dextrose (YEPD) medium was used for the adaptive cultivation of *S*. *cerevisiae*. Adaptive laboratory evolution (ALE) was performed by transferring the culture to fresh YEPD medium with a gradient increase in the concentration of 2-PE as an inhibitor. Briefly, *S*. *cerevisiae* was cultured in YEPD liquid medium at 30°C and 200 r/min for 24 h. Subsequently, the culture was transferred into YEPD liquid medium containing 1 g/L of 2-PE at an inoculum ratio of 2% (v/v) for another 24 h. Next, the cell broth was spread on a YEPD medium plate containing 1 g/L of 2-PE and cultured at 30°C for 48 h. Every yeast colony was picked and transferred to a 24-well plate containing 3 mL of YEPD medium and 1 g/L of 2-PE, cultured at 30°C and 200 r/min for 24 h. Three replicates were set for each colony. The density of yeast cells that reached an OD_600_ of 8 within 24 h of cultivation under 2-PE stress was further screened for ALE experiments. ALE was performed by gradually increasing the concentrations of 2-PE in the culture until the density of yeast cells could not reach an OD_600_ of 8 within 24 h of cultivation. For each strain that could tolerate different 2-PE concentrations, glycerol stocks were made at each concentration of evolved populations.

### 2.3 DNA manipulation and vector construction

The codon-optimized phenylpyruvate decarboxylase *kdcA* gene from *Lactococcus lactis* (*llkdcA*) and the L-glutamate oxidase genes from *Streptomyces sp*. X-119-6 (*strlgox*) and *Kitasatospora setae* KM-6054 (*kitlgox*) were synthesized and ligated with the *TPI* promoter and *TT* terminator via fusion PCR, resulting in the *P*_*TPI*_*-llkdcA-T*_*TT*_, *P*_*TPI*_*-strlgox-T*_*TT*_ and *P*_*TPI*_*-kitlgox-T*_*TT*_ fragments, respectively. The codon-optimized *tyrB* gene from *E*. *coli* (*ectyrB*) was amplified and inserted with the *TPI* promoter and *TT* terminator by ligation, resulting in the pYX-*P*_*TPI*_*-ectyrB-T*_*TT*_ overexpression vector. The *ARO8*, *ARO9*, *ARO10* and *ADH2* wild-type genes were amplified from the genomic DNA of *S*. *cerevisiae* CICC31906. Multiple enzymes were overexpressed in fusion protein with linkers (GGGGS) between them. The genes were amplified into fragments equipped with linkers and connected together by overlap PCR. All of these gene fragments were amplified by PCR using the primers and templates described in S1 and S2 Tables. Then, the DNA fragments were gel-purified, cloned into the pMD-19T vector for sequence confirmation and then cloned into the pYX212 vector under the *TPI* promoter with the *TT* terminator for overexpression.

Yeast cell transformation was carried out via the lithium acetate method. The transformants were selected on YEPD medium with 350 μg/mL G418.

### 2.4 Gene disruption

Gene deletion yeast strains were constructed by iterative replacement of the targeted genes by the Cre/loxp method. The upstream and downstream gene fragments were amplified using *S*. *cerevisiae* CICC31906 genomic DNA as a template. The linearized expression cassette G418MX was constructed by ligating the upstream, G418MX, and downstream fragment genes via fusion PCR and gel purification. After the plasmid and oligonucleotide were simultaneously transformed into *S*. *cerevisiae* and selected on a YEPD plate with G418, mutants were confirmed by PCR from genomic DNA preparations and finally selected. The primers and templates used to target the genes are indicated in S3 Table.

### 2.5 qPCR analysis

The yeast cells were harvested after 24 h of culture. Total RNA was extracted using the Yeast Total RNA Isolation Kit by Sangon Biotech (Shanghai, China) following the manufacturer’s instructions. The relative expression levels were determined by performing real-time quantitative PCR using *scACT1* as a reference gene. Primers for qPCR analysis are indicated in S4 Table. Two hundreds nanograms of RNA was reverse transcribed by a High Capacity cDNA Reverse Transcription Kit (Takara, Shiga, Japan) and quantified in triplicate by TB Green *Premix Ex Taq* II (Takara, Shiga, Japan) using the primers listed in S4 Table. Reactions were performed on the Viia 7 Real Time PCR Instrument (Thermo Fisher Scientific, MA, USA), and data were analyzed using the Viia 7 Software. All analyses were performed in triplicate.

### 2.6 Strain cultivation

Yeast strains were cultivated at 30°C in YEPD medium or yeast synthetic minimal (YSM) medium. YEPD medium is composed of 20 g/L glucose, 10 g/L yeast extract and 20 g/L peptone. YSM medium is composed of 20 g/L glucose, 6.7 g/L L-phenylalanine, 4 g/L yeast extract, 3 g/L KH_2_PO_4_, 0.5 g/L MgSO_4_ and 1 mL/L trace element solution, adjusting the pH with KOH to 6.0. YSM light medium is composed of 20 g/L glucose, 4 g/L L-phenylalanine, 1 g/L yeast extract, 3 g/L KH_2_PO_4_, 0.5 g/L MgSO_4_ and 1 mL/L trace element solution, adjusting the pH with KOH to 6.0. The trace element solution contained 0.05 g/L CuSO_4_·5H_2_O, 2 g/L FeSO_4_·7H_2_O, 0.2 g/L MnCl_2_·4H_2_O, 2 g/L CaCl_2_·2H_2_O, and 0.5 g/L ZnCl_2_ dissolved in 2 mol/L HCl and was stored at 4°C. Cloning and plasmid propagation were carried out using the *E*. *coli* strain JM109, which was grown at 37°C in super optimal broth (SOB) medium with 100 μg/mL ampicillin. All strains were cultivated with constant orbital shaking at 200 rpm. All the strains were stored at -80°C in 15% glycerol.

To initiate the experiment, the yeast strains were grown in YEPD medium for 16–20 h at 30°C and 200 rpm in rotary shakers to reach a log phase OD_600_ of 8. Then, shake-flask fermentations were carried out in 250-mL Erlenmeyer flasks containing 50 mL YSM medium inoculated with 5% (v/v) seed cultures at 30°C and 200 rpm in rotary shakers. In addition, 20 g/L glucose was added if necessary. The fermentation experiments were performed in triplicate.

### 2.7 Quantification and analysis of metabolites

#### Dry cell weight

The cell density was measured by the absorbance under 600 nm (OD_600_) with UV-2100 UV/Vis spectrophotometer (UNICO Instruments Co., Shanghai, China). The cell concentration was calculated from a standard curve relating OD_600_ to dry cell weight (DCW) using the following formula: 1 OD_600_ = 0.3 g DCW/L.

#### High-performance liquid chromatography methods

The culture broth was centrifuged at 13,500 × g for 2 min, and the supernatant was analyzed for aromatic compound concentrations using a Dionex Ultimate 3000 HPLC (Thermo Fisher Scientific, MA, USA) equipped with a XBridge BEH C18 250 mm×4.6 mm column (particle size 5 μm) (Waters, MA, USA). L-phenylalanine, phenylacetate, 2-phenylethanol and phenylpyruvate samples were analyzed with 70% solvent A (water with 0.1% formic acid) and 30% solvent B (acetonitrile) at a flow rate of 0.5 mL/min with UV detector under 210 nm. L-phenylalanine was detected at 7.5 min, phenylpyruvate at 3.5 min, 2-PE at 13.3 min and phenylacetate at 5.3 min. All the peak areas of these aromatic compounds were integrated and used for quantification by fitting with standard curves. Phenylacetate was not detected in all the samples of strains, so that the results of phenylacetate were omitted.

The quantitation of glucose, ethanol, glycerol and acetate were determined by a Dionex Ultimate 3000 HPLC (Thermo Fisher Scientific, MA, USA) equipped with an Aminex HPX-87G column (Bio-Rad, CA, USA). The column was eluted with 5 mM H_2_SO_4_ at a flow rate of 1.0 mL/min at 50°C for 22 min. Glucose, ethanol, acetate and glycerol were detected by RI detector at 9.6 min, 17.9 min, 14 min and 12.9 min, respectively. For all samples, at least three biological replicates were analyzed.

### 2.8 Statistical analysis

The statistical analysis was performed using SPSS version 20.0 (SPSS Inc., IL, USA) for the one-way analysis of variance (ANOVA) and means were compared using Student’s t-test (one-tailed; two-sample unequal variance). The probability value (*p*-value) of < 0.05 was considered significant.

## 3 Results

### 3.1 Adaptive evolution of *S*. *cerevisiae* for tolerance of 2-PE

**Fig 2:**
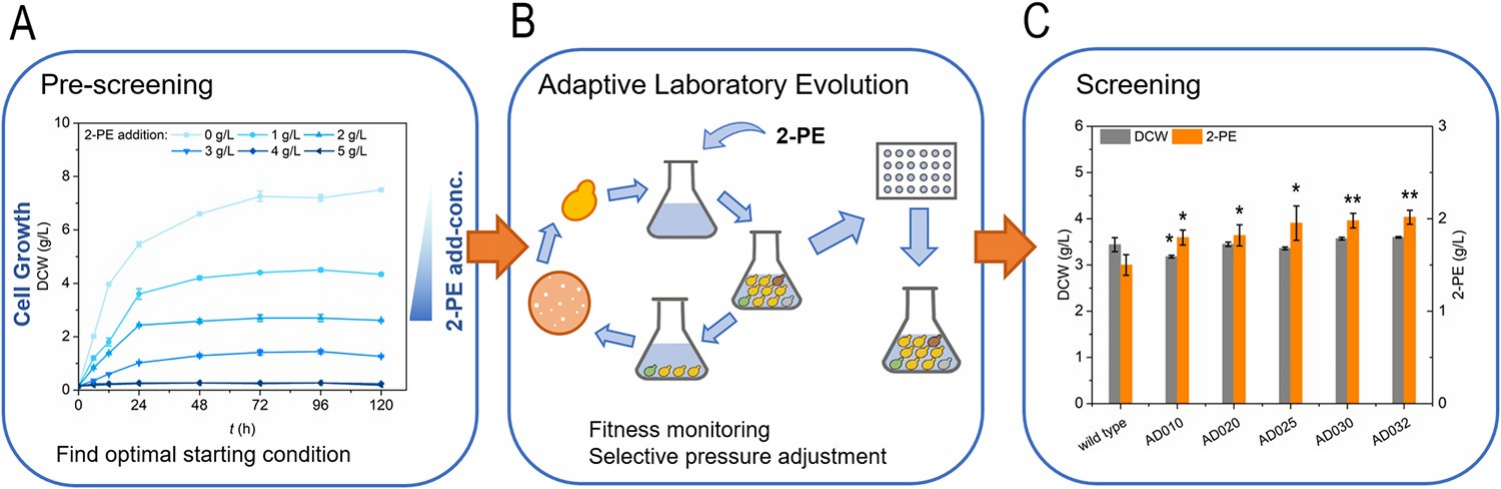
Overview of the experimental procedures used to obtain 2-PE tolerant yeast strain. (A) Cell growth of the control strain WT-A in YEPD medium added with different concentration of 2-PE addition. (B) Schematic diagram of the adaptive laboratory evolution process of *S*. *cerevisiae* strain AD032. (C) Cell growth and 2-PE titer of ALE stains in YSM light medium. Data are presented as the averages of the results of three independent experiments. Error bars show standard deviations. Error bars show standard deviations. Statistical analysis was performed by using Student’s t-test (one-tailed; two-sample unequal variance; *p < 0.05, **p < 0.01, ***p < 0.001).

To avoid the use of extractants during the fermentation process, tolerance to 2-PE needs to be improved through ALE methods. Prior to the experiment, an initial growth test was conducted using *S*. *cerevisiae* WT-A. The inhibitory concentration of 2-PE to this yeast strain was identified after the addition of 0 to 5 g/L 2-PE to YEPD medium (Fig 2A). Strain WT-A showed a negative correlation between biomass yield and 2-PE concentration in the medium. To facilitate selection of a mutant with improved tolerance of 2-PE fermentation, *S*. *cerevisiae* WT-A was subjected to an adaptive evolutionary process, as shown in Fig 2B. During the evolutionary process, the growth was monitored and the concentration of 2-PE progressively increased. This promoted a continuous improvement in the tolerance fitness (maximum growth rate), while using an approach similar to that previously described [28]. As shown in Fig 2C, the yeast cells were cultivated in a YEPD medium, with a gradual increase in 2-PE levels in the medium from 1 g/L to 3.2 g/L during serial subculture. Approximately 16 generations evolved in the process. Finally, strain AD032, which could grow in the medium with 3.2 g/L 2-PE, was selected for further characterization.

**Fig 3:**
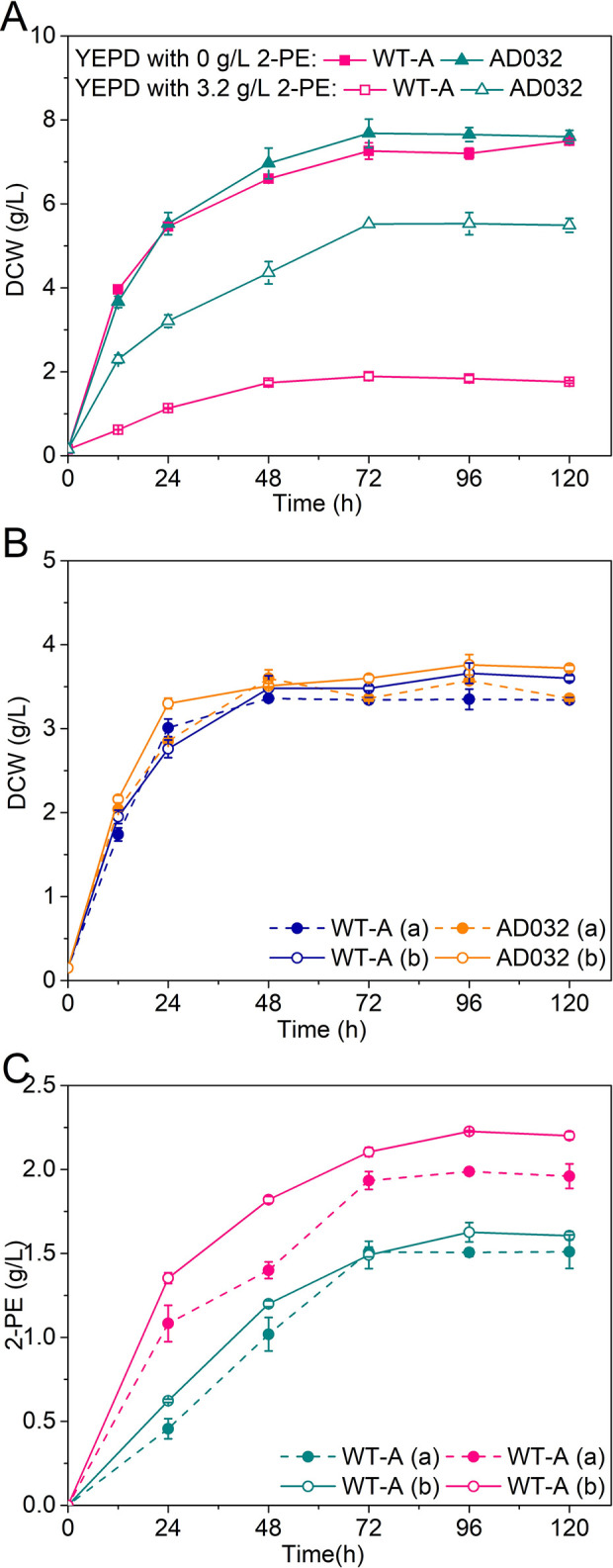
Improvement of cell growth and 2-PE production by adaptive laboratory evolution of *S*. *cerevisiae* strain AD032. (A) Tolerance of the adaptive strain AD032 and control strain WT-A under 2-PE stress in YEPD medium. (B) Cell growth of control strain WT-A and evolutionary adaptive strain AD032 in the flask fermentation with 4 g/L phenylalanine (a) and 6.7 g/L phenylalanine (b) culture medium. (C) Improvement of 2-PE production of control strain WT-A and evolutionary adaptive strain AD032 in the flask fermentation with 4 g/L phenylalanine (a) and 6.7 g/L phenylalanine (b) culture medium. Data are presented as the averages of the results of three independent experiments. Error bars show standard deviations.

The tolerance of the adaptive strain AD032 and control strain WT-A under 2-PE stress in YEPD medium was studied, as shown in Fig 3A. All the yeast strains grew well in YEPD medium without 2-PE, with a biomass of approximately 7 g/L DCW. With 3.2 g/L of 2-PE added to YEPD medium, the growth of strain WT-A was merely 1.89 g/L after 120 h of cultivation. In contrast, the biomass of the adaptive strain AD032 was 5.62 g/L of DCW. The 2-PE fermentation performance of *S*. *cerevisiae* AD032 was evaluated in shake flasks containing YSM medium. The discrepancy of cell growth between strains WT-A and AD032 was minimal; however, AD032 showed a 2.33-fold increase in 2-PE production as shown in Figs 3B, 3C and S1. The improvement in cell growth, as well as the ability to synthesize 2-PE by AD032, was attributed to yeast optimization at genetic background level under high concentrations of 2-PE stress. This was confirmed by complete genome sequencing; further studies by our laboratory are still in progress (data not shown).

### 3.2 Synthesis of 2-PE based on fusion protein via the Ehrlich pathway

A feeding experiment was carried out to determine the optimal enzyme combination for 2-PE production. Considering its easier commercial availability, L-phenylalanine was chosen as the substrate for the bioconversion study [29]. In *S*. *cerevisiae*, phenylpyruvate is an important intermediate in 2-PE biosynthesis. Three phenylalanine transferases, TyrB from *E*. *coli*, which showed prominent performance in L-phenylalanine synthesis [30], together with Aro8 and Aro9 from *S*. *cerevisiae*, were selected as candidates to catalyze the reversible transformation between phenylpyruvate and L-phenylalanine for the first step. Considering that the enzymatic ability of phenylpyruvate decarboxylase was dominant in the synthesis of 2-PE [31], Aro10 from *S*. *cerevisiae* and KdcA from *L*. *lactis* [32] were chosen for their intensive activities instead of KivD, another 2-oxo acid decarboxylase from *L*. *lactis* [33]. In addition, overexpression of aldehyde dehydrogenase Adh2p could improve 2-PE production [26], *ADH2* was co-expressed along with the phenylpyruvate decarboxylase and phenylalanine transferases in this study.

**Fig 4:**
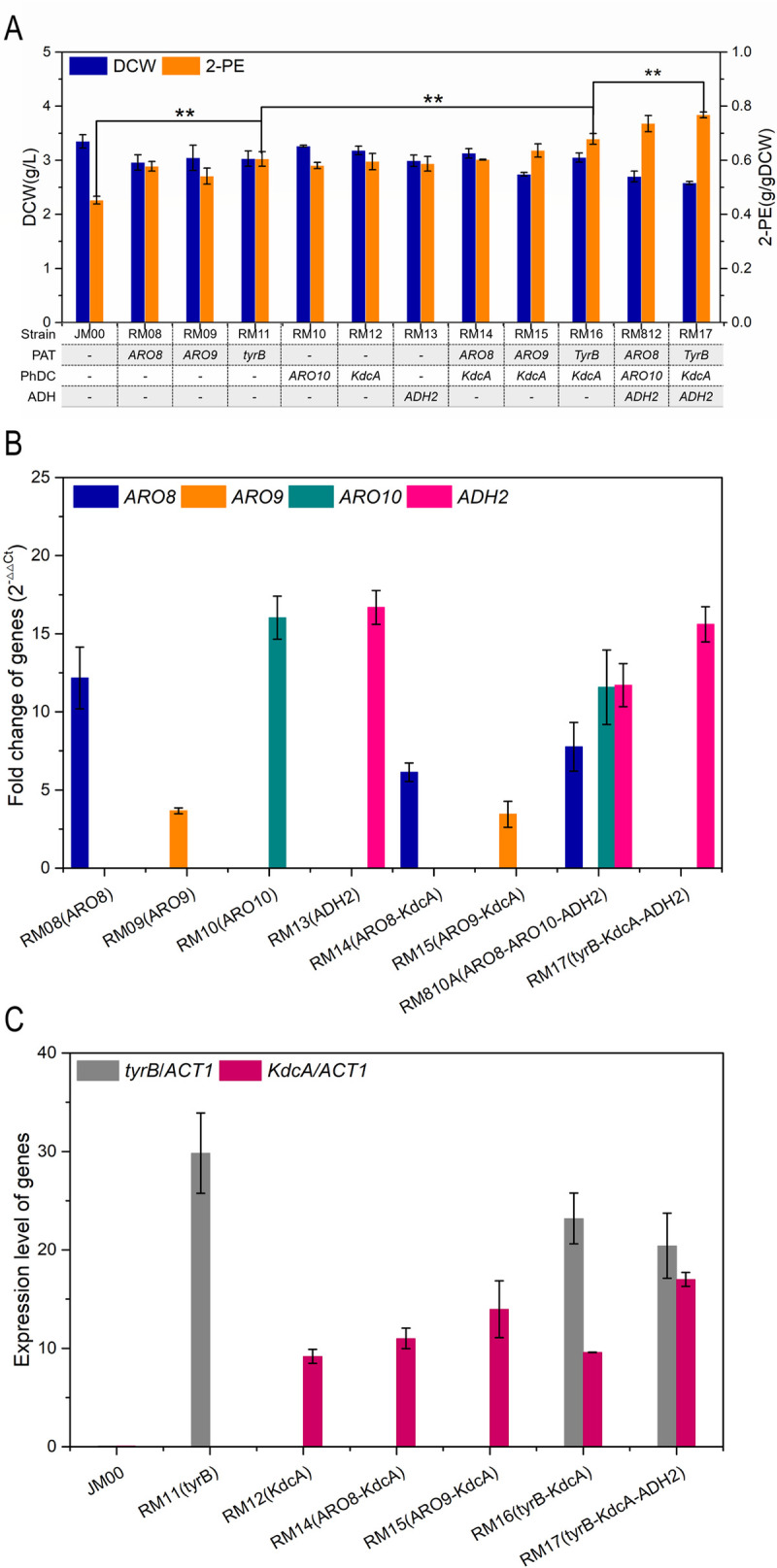
Effective expression of individual key enzyme and fusion protein in Ehrlich pathway. (A) Influence on cell growth and 2-PE production by overexpressing enzymes of Ehrlich pathway respectively. The gene types were listed under the bar graph. Enzyme abbreviations: PAT, phenylalanine transaminase; PhDC, phenylpyruvate decarboxylase; ADH, alcohol dehydrogenase. The mutant strains were cultured in YSM light medium (4 g/L phenylalanine) for flask fermentation. (B) Gene *ARO8*, *ARO9*, *ARO10* and *ADH2* transcription levels in the mutant strains. (C) Gene *tyrB* and *KdcA* transcription levels in the mutant strains. Data are presented as the averages of the results of three independent experiments. Error bars show standard deviations. Statistical analysis was performed by using Student’s t-test (one-tailed; two-sample unequal variance; *p < 0.05, **p < 0.01, ***p < 0.001).

**Fig 5:**
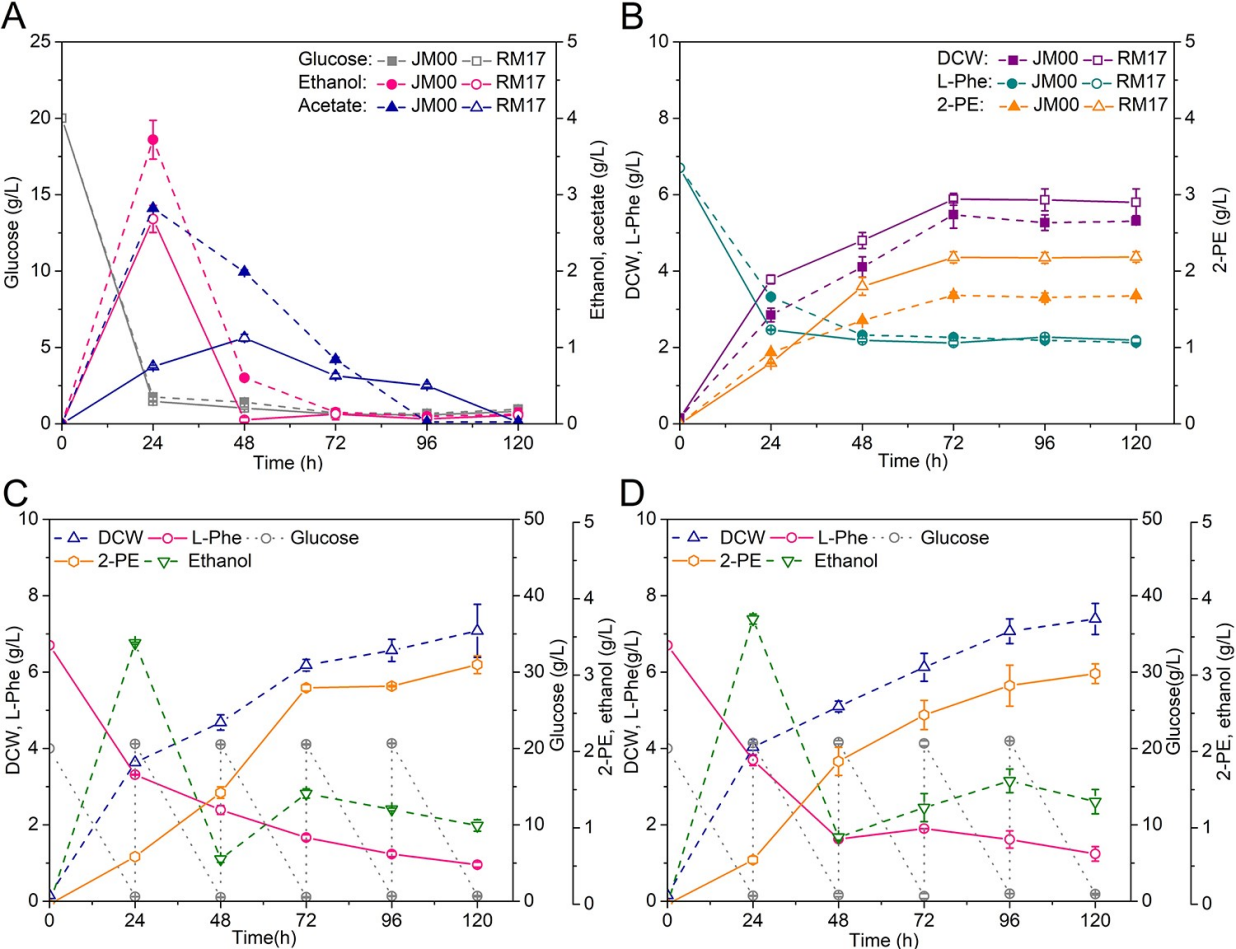
Influence on 2-PE production by fusion-expressing mutant strains. (A) Glucose consumption, ethanol and acetate of JM00 and RM17 (WT-A *P*_*TPI*_*-ectyrB-llkdcA-scADH2*) in YSM medium. (B) Cell growth, 2-PE titers and L-phenylalanine consumption of JM00 and RM17 in YSM medium. (C) Fermentation view of RM17 in YSM medium with the addition of 20 g/L glucose every 24 h. (D) Fermentation view of RM812 (WT-A *P*_*TPI*_*-scARO8-scARO10-scADH2*) in YSM medium with the addition of 20 g/L glucose every 24 h. Data are presented as the averages of the results of three independent experiments. Error bars show standard deviations.

All mutants with the overexpression vector exhibited superior 2-PE production (Fig 4A). The gene expression levels were confirmed via mRNA levels in the overexpression strains, as shown in Fig 4B and 4C. Under YSM light medium flask fermentations, expressing genes *tyrB*, *kdcA* and *ADH2*, 0.60 g/g DCW, 0.59 g/g DCW and 0.59 g/g DCW of 2-PE was produced, respectively. The production shows, a 1.34-fold, 1.32-fold and 1.32-fold increase compared to that of strain JM00, respectively. Therefore, we chose these enzymes for fusion protein expression, which may improve catalytic efficiency through the Ehrlich pathway. The function of the fusion protein was confirmed by changing the 2-PE titers through the step-by-step expression of the enzymes, displayed as mutants RM11, RM16, and RM17 in Fig 5A. After confirmation of its effectiveness, the fusion protein was expressed in mutant RM17 and then cultured in YSM medium, resulting in 2-PE accumulation of 2.18 g/L, which was 1.30-fold higher than that of JM00 (Fig 5B). Furthermore, an increase in ethanol production and L-phenylalanine use in strain RM17 was observed, compared to strain JM00 (Fig 5A and 5B). Through the addition of glucose (20 g/L every 24 h) to the YSM, the mutant strain reached 7.08 g/L DCW and produced 3.14 g/L 2-PE, with a better use of L-phenylalanine after 120 h flask fermentation, as shown in Fig 5C. The mutant RM812 (WT-A *P*_*TPI*_*-scARO8-scARO10-scADH2*) produced 3.02 g/L 2-PE (Fig 5D).

### 3.3 Enhanced α-ketoglutarate supplementation by *lgox*-expression to improve 2-PE production

**Fig 6:**
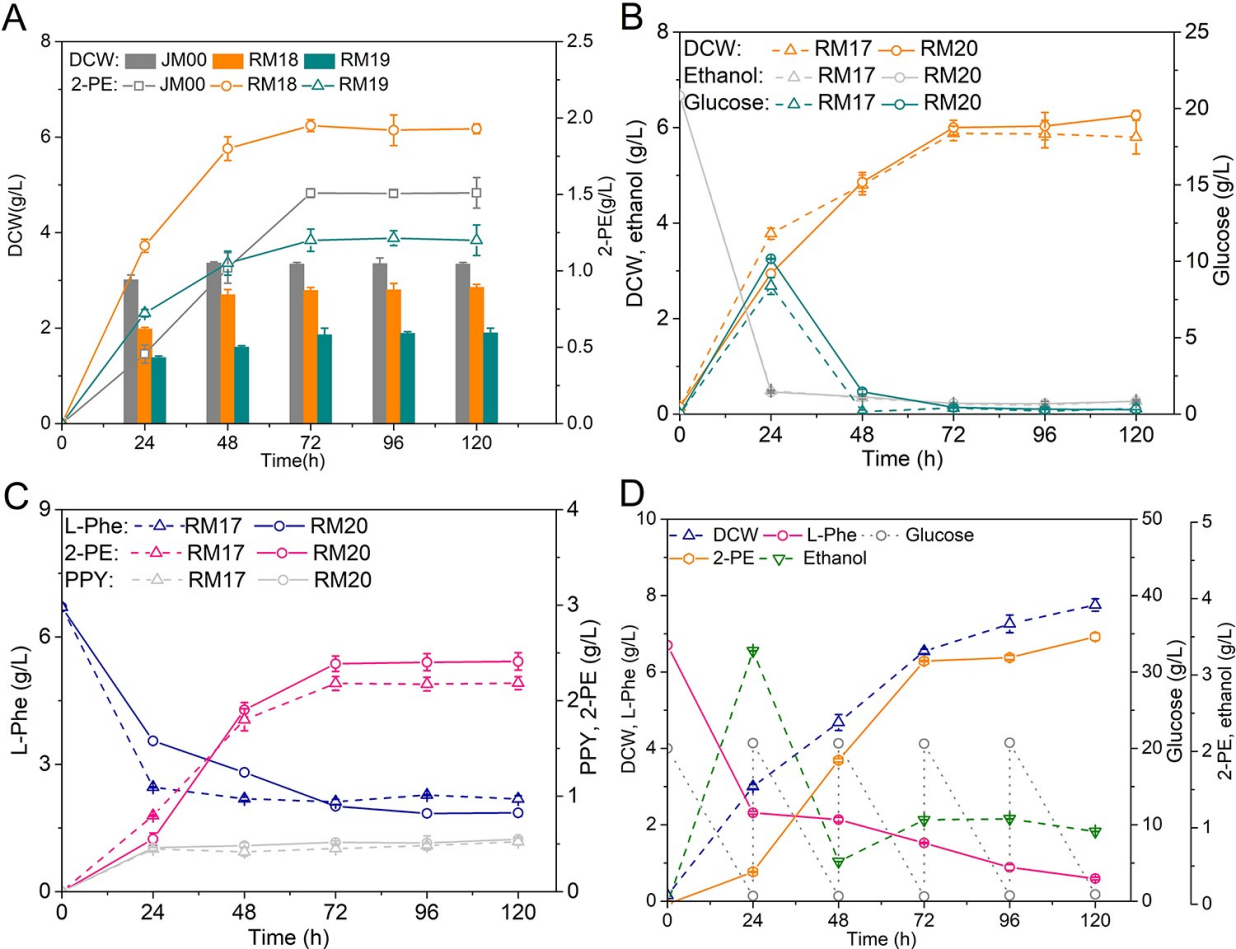
The new expression module of *lgox* for 2-PE synthesis in Ehrlich pathway. (A) Influence on cell growth and 2-PE production by L-glutamate oxidase expressing mutant strains. (B) Cell growth, ethanol and glucose consumption of RM17 (WT-A *P*_*TPI*_*-ectyrB-llkdcA-scADH2*) and RM20 (WT-A *P*_*TPI*_*-ectyrB-llkdcA-scADH2 P*_*TPI*_*-strlgox*) in YSM medium. (C) Improvement of 2-PE synthesis and L-phenylalanine consumption in RM20 compared to RM17 in YSM medium. (D) Fermentation view of RM20 in YSM medium with the addition of 20 g/L glucose every 24 h. Data are presented as the averages of the results of three independent experiments. Error bars show standard deviations.

The conversion of L-phenylalanine to phenylpyruvate requires α-ketoglutarate as an amino receptor, after which L-glutamate is generated. To promote the metabolic fluxes from L-phenylalanine to phenylpyruvate, the supply of the amino receptorα-ketoglutarate should be improved. Although *S*. *cerevisia*e was demonstrated to be capable of carrying out the reaction from L-glutamate to α-ketoglutarate by L-glutamate oxidase (EC 1.4.3.11, encoded by *lgox*), the activity of L-glutamate oxidase was low [34,35]. In the overexpression mutant, α-ketoglutarate may be lacking in the 2-PE synthetic process, and the original low activity of L-glutamate oxidase in yeast probably could not provide enough α-ketoglutarate from L-glutamate. Based on recent studies of the activity and properties [35], L-glutamate oxidases from *Streptomyces sp*. X-119-6 and *Kitasatospora setae* KM-6054 were chosen to overexpress for 2-PE production (Fig 6A). The results shown in Fig 6A indicate that the strain RM18 (WT-A *P*_*TPI*_-*strlgox*) exhibited a slightly decreased dry cell weight of 2.85 g/L but a significantly increased 2-PE titer of 1.95 g/L compared to JM00 (*p*<0.05). While production of 2-PE in the strain RM19 (WT-A *P*_*TPI*_-*kitlgox*) decreased to 1.21 g/L.

Therefore, the gene cassette *P*_*TPI*_-*strlgox* was introduced into RM17, resulting in strain RM20 (WT-A *P*_*TPI*_*-ectyrB-llkdcA-scADH2 P*_*TPI*_*-strlgox*). By culturing in YSM medium, RM20 reached a 2-PE accumulation of 2.41 g/L, which was 1.11-fold than RM17 (Fig 6C), indicating that overexpression of *strlgox* provides important assistance in 2-PE synthesis. It is also worth noting that the utilization of L-phenylalanine by RM20 was more efficient, and more phenylpyruvate was produced compared to RM17 (Fig 6C), which could be regarded as an effect of *strlgox* expression. Furthermore, through the addition of glucose (20 g/L every 24 h), the mutant strain reached 7.75 g/L dry cell weight and produced 3.50 g/L 2-PE with better use of L-phenylalanine after 120 h of flask fermentation in YSM medium, as shown in Fig 6D.

### 3.4 Improvement of 2-PE production by deleting phenylpyruvate decarboxylase Pdc5

**Fig 7:**
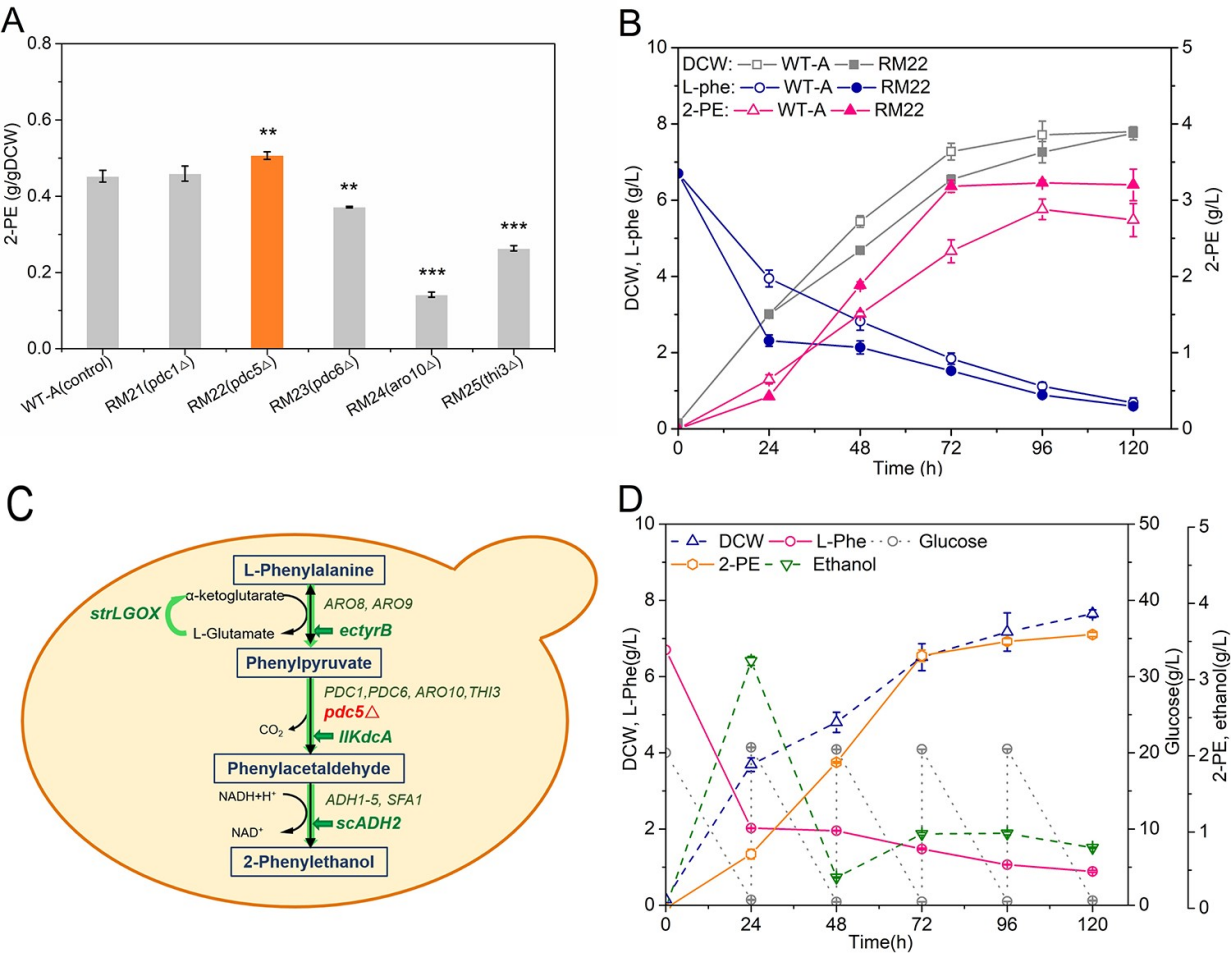
Impact on 2-PE synthesis in *PDC5* deleting mutant. (A) Influence on 2-PE synthesis abilities of yeast cell by mutant strains RM21 (WT-A *pdc1*Δ), RM22 (WT-A *pdc5*Δ), RM23 (WT-A *pdc6*Δ), RM24 (WT-A *aro10*Δ), and RM25 (WT-A *thi3*Δ). (B) Fermentation view of RM22 (WT-A *pdc5*△). (C) Diagram of the genetic methods in the engineered strain RM26. (D) Fermentation view of RM26 (WT-A *pdc5*△ *P*_*TPI*_*-ectyrB-llkdcA-scADH2 P*_*TPI*_*-strlgox)* in YSM medium with the addition of 20 g/L glucose every 24 h. Data are presented as the averages of the results of three independent experiments. Error bars show standard deviations. Statistical analysis was performed by using Student’s t-test (one-tailed; two-sample unequal variance; *p < 0.05, **p < 0.01, ***p < 0.001).

The main function of pyruvate decarboxylase (Pdc1, Pdc5, Pdc6) in *S*. *cerevisiae* is to catalyze the reaction from pyruvate to acetaldehyde; these enzymes also showed phenylpyruvate decarboxylase activity during 2-PE synthesis, similar to Aro10. As a eukaryotic organism with a mature regulatory mechanism, *S*. *cerevisiae* has a complex and strict self-control system in metabolic pathways. For instance, the expression level of gene *PDC5* strongly increased in the *PDC1*-absent mutant and was transcriptionally induced in response to thiamin starvation [36,37]. Accordingly, individual phenylpyruvate decarboxylase-deleted strains were constructed to analyze the influence on 2-PE production, resulting in the mutant strains RM21 (WT-A, *pdc1*Δ), RM22 (WT-A, *pdc5*Δ), RM23 (WT-A, *pdc6*Δ), RM24 (WT-A, *aro10*Δ), and RM25 (WT-A, *thi3*Δ). As shown in Fig 7A, the deletion of gene *ARO10* resulted in a decrease of 2-PE production to 0.14 g/g DCW in the YSM light medium. Remarkably, we found that the deletion of the *PDC5* gene alone increased the 2-PE-producing capability of the yeast to 0.51 g/g DCW, which was higher than that of the 0.45 g/g DCW in the control strain WT-A. The mutant strain RM22 (WT-A, *pdc5*Δ) was cultured in YSM medium with the addition of 20 g/L glucose every 24 h. As shown in Fig 7B, the mutant strain RM22 reached 7.80 g/L DCW and produced 3.23 g/L 2-PE after 120 h. Therefore, deleting the gene *PDC5* could contribute to the synthesis of 2-PE in this cultivate condition. Considering of this, the metabolic modules were assembled in the yeast strain WT-A for further improvement of 2-PE production (Fig 7C). Based on the mutant yeast strain RM20, RM26 (WT-A *pdc5*Δ *P*_*TPI*_*-ectyrB*-*llkdcA*-*scADH2 P*_*TPI*_*-strlgox*) was constructed for further 2-PE improvement. RM26 was cultured in YSM medium supplemented by 20 g/L glucose every 24 h, reaching 7.82 g/L DCW and producing 3.23 g/L 2-PE with improved use of L-phenylalanine after 120 h (Fig 7D).

### 3.5 Analysis of the influence of condition factors on the fermentation process

**Fig 8:**
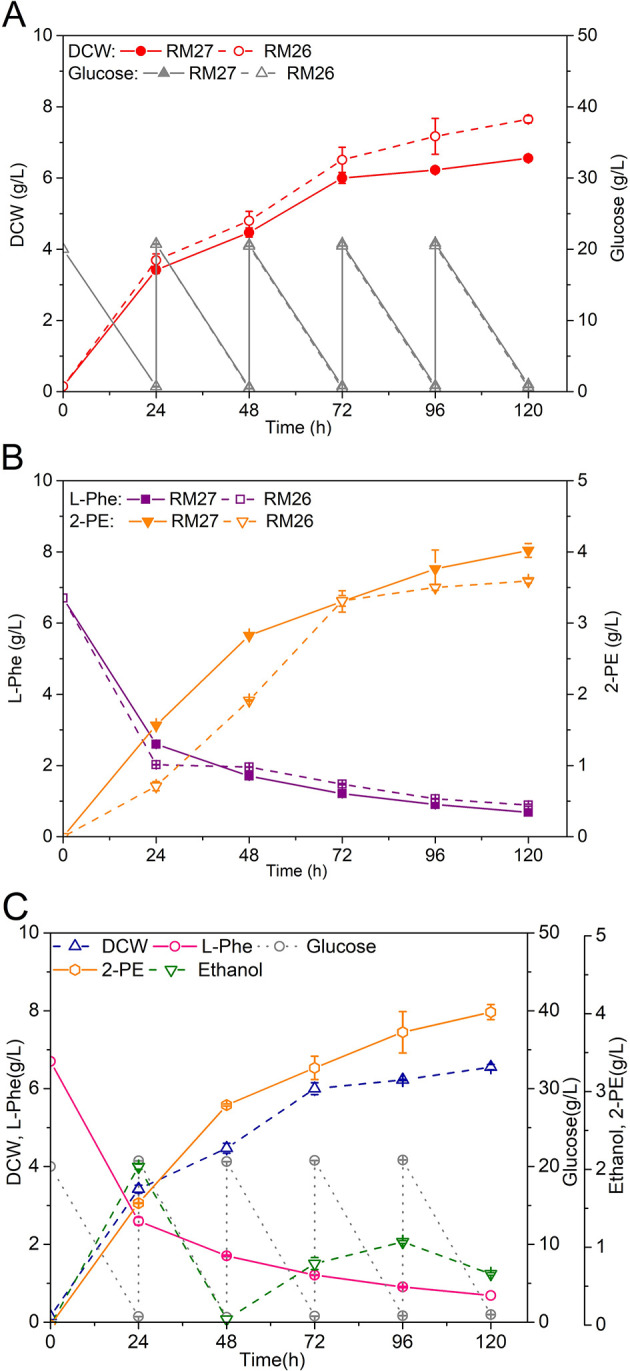
Fermentation view of yeast strains RM26 and RM27. (A) Cell growth, ethanol and glucose consumption of RM26 and RM27 in YSM medium with the addition of 20 g/L glucose every 24 h. (B) Improvement of 2-PE synthesis and L-phenylalanine consumption of RM26 and RM27 in YSM medium with the addition of 20 g/L glucose every 24 h. (C) Fermentation view of RM27 (AD032 *pdc5*△ *P*_*TPI*_*-ectyrB-llkdcA-scADH2 P*_*TPI*_*-strlgox)* in YSM medium with the addition of 20 g/L glucose every 24 h.

**Fig 9:**
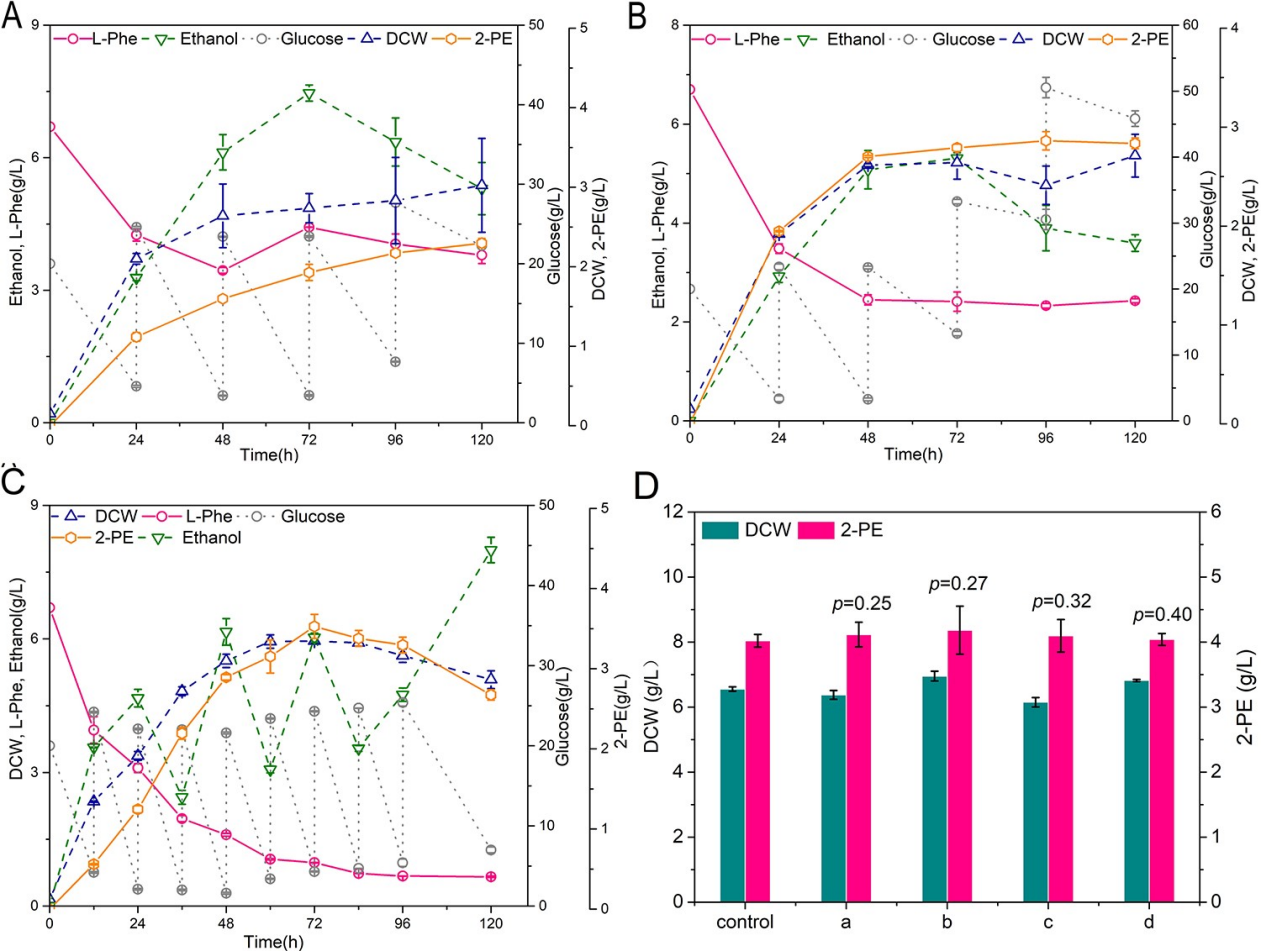
Optimization of 2-PE synthesis in metabolic engineered yeast strain RM27. (A) Fermentation view of JM00 with L-phenylalanine as the sole nitrogen. (B) Fermentation view of RM27 with L-phenylalanine as the sole nitrogen. (C) Fermentation view of RM27 in YSM medium with the addition of glucose every 12 h. (D) Fermentation view of RM27 in YSM medium with the addition of L-glutamate or α-ketoglutarate. (a) One mmol/L α-ketoglutarate was added to the medium at 24 h. (b) One mmol/L α-ketoglutarate was added to the medium every 24 h. (c) One mmol/L L-glutamate was added to the medium at 24 h. (d) One mmol/L L-glutamate was added to the medium every 24 h. Data are presented as the averages of the results of three independent experiments. Error bars show standard deviations. Statistical analysis was performed by using Student’s t-test (one-tailed; two-sample unequal variance; *p < 0.05, **p < 0.01, ***p < 0.001).

Due to the yeast strain that produced high 2-PE production may be under the inhibition [38,39], all valid modules were combined into the adaptive evolutionary strain AD032, resulting mutant strain RM27 (AD032 *pdc5*Δ *P*_*TPI*_*-ectyrB*-*llkdcA*-*scADH2 P*_*TPI*_*-strlgox*). The performances of strain RM26 and RM27 cultured in YSM medium with 24-h interval supplementation of glucose are shown in Fig 8A and 8B. The results of 2-PE titers cultured in YSM medium containing different concentrations of glucose are shown in Figs 8C and 9C. The 12-h interval supplementation of glucose was unfavorable for 2-PE synthesis, which may have led to the generation of more by-products (Fig 9C). The optimal initial concentration of glucose was approximately 20 g/L; however, it would have been exhausted within 24 h (data not shown). Therefore, 20 g/L glucose was added to the culture medium every 24 h, which provided a carbon source to improve the growth-coupling synthesis of 2-PE. Therefore, RM27 was cultured, with the fermentation performance shown in Fig 8C. The 2-PE titer reached 4.02 g/L at 120 h, with thorough use of L-phenylalanine. When cultured with L-phenylalanine as the sole nitrogen provider, the yeast strain RM27 produced only 2.88 g/L 2-PE with poor cell growth (Fig 9A and 9B). Additionally, transamination may affect the synthesis of 2-PE when there is a lack of supply of L-glutamate or α-ketoglutarate, even if L-glutamate oxidase is overexpressed. Considering this, L-glutamate and α-ketoglutarate were added to the medium respectively as shown in Fig 9D. The results illustrated that the addition of L-glutamate or α-ketoglutarate after 24 h of shake-flask culture did not significantly change 2-PE production (*p*>0.05). This suggests that in the *strlgox*-overexpression strain, the L-glutamate and α-ketoglutarate produced by yeast can be completely used within equilibrium during the transamination for 2-PE synthesis, with no need for additional supply.

## 4 Discussion

The method of ALE was a moderate evolutionary approach by which more forward mutations could be obtained for further study. Previous studies applying ALE have evolved in favor of various *S*. *cerevisiae* phenotypes, such as tolerance to inhibiting concentrations of organic acids [28,40], stress resistance [41] and high temperature [42]. In our study, ALE was used to guarantee yeast cell growth under high 2-PE concentration stress and to enhance the synthesis of 2-PE production. With reference to this we follow the method of Pereira et al. [28], due to the similar objective of improving the resistance to compounds that are harmful to yeast cells. Improving the tolerance of 2-PE in the *S*. *cerevisiae* strain markedly enhanced the titer of 2-PE biotransformation from 1.51 g/L to 2.01 g/L. Considering further metabolic engineering, this method appeared to be more moderate than other mutagenesis of strains, resulting in a relatively uncomplicated genetic background. Yeast strain tolerance induced by 2-PE stress involves many upregulated genes related to mitochondrial cytoplasmic, and plasma membrane proteins [9]. However, further studies should be conducted on the mechanism of cell tolerance to 2-PE. As we focus on the application of 2-PE in the food industry, stringent demand for the availability of food grade certification in the fermentation process should be guaranteed. The platform strain AD032, proposed in this study by the adaptive evolutionary method, effectively increased the yield of 2-PE using generally regarded as safe certified yeast. Instead of introducing organic solvents, which are a food-safety substandard for product purification, our platform strain showed potential merits for simplifying the process, equipment, and maintenance of food grade in the fermentation process. Based on the genomic and transcriptome analysis of the evolutionary strain AD032, the mechanism of 2-PE tolerance, which is completely unclear, will be studied in our future work.

To accomplish the production of 2-PE, we assembled a synthetic pathway composed of distinct enzymes from different origins in the Ehrlich pathway. Among these, KdcA showed a superior Vmax/km ratio of enzymatic modification than Aro10, as well as higher activity for phenylpyruvate [33]. Notably, L-phenylalanine in the culture medium caused upregulation of 2-oxo acid decarboxylases, which have stronger substrate specificity [33], resulting in better 2-PE synthesis performance by *kdcA*-expression as shown in Fig 3A. Considering the possible decrease in the expression level and activity of the fused protein, we used the (-GGGGS-) linker between every enzyme to maintain enzyme activity or stability. On the other hand, although the by-product phenylacetate was not detected in either the control or the engineered strains, possible side reactions should be prevented by linker insertion due to the proximity effect of the fusion enzymes. However, compared to the conversion of L-phenylalanine and phenylpyruvate, the reaction of α-ketoglutarate to L-glutamate in phenylalanine transamination, which provides an amino receptor, has always been ignored. Considering this, L-glutamate oxidase, which was found to be the best catalyst to oxidize the L-glutamate amino group to a ketonic group without the need for an exogenous cofactor [34], efficiently promoted the synthesis of 2-PE. In this study, L-glutamate oxidase from *Streptomyces sp*. X-119-6 was first used to promote transamination for the production of 2-PE in yeast. Through the co-expression of L-glutamate oxidase and phenylalanine aminotransferase, a system for recovering ketoglutaric acid for the conversion of ammonia to alcohol synthesis was developed. Furthermore, the addition of L-glutamate to the fermentation culture medium did not obviously affect the 2-PE titer, indicating that the activity of L-glutamate oxidase is sufficient to support the requirement for the transamination reaction in the Ehrlich pathway. The L-glutamate oxidase from *Streptomyces* was effectively expressed in yeast and promoted the synthesis of 2-PE as expected. However, overexpressing L-glutamate oxidase from *Kitasatospora setae* in *S*. *cerevisiae* showed a decrease in 2-PE production than the control strain, which indicated functional-expressed but negative effect on 2-PE synthesis. The significant decrease in 2-PE was probably due to the diversity of L-glutamate oxidases from different strains, which could not be accurately predicted without further study of the catalytic mechanism. On the other hand, pyruvate decarboxylase, a key enzyme in alcoholic fermentation in *S*. *cerevisiae*, can degrade pyruvate to acetaldehyde. Pdc5 was reported to catalyze the decarboxylation of phenylpyruvate [43], but the impact of *PDC5* deletion on 2-PE synthesis is still unclear. Li showed that deletion of *PDC5* in a strain carrying overexpressed *ILV2* and *ARO10* resulted in 8-fold higher isobutanol productivity in *S*. *cerevisiae* [44]. Similarly, we found 2-PE yield was increased in the *pdc5*-absent strain RM22 and made use of this for improved 2-PE production in the metabolic strategies in this study. Nevertheless, further research is needed on whether *PDC5* has a role of regulatory factor, which may make a considerable contribution to the synthesis of all aromatic compounds in yeast.

As it is widely used in the food industry, the demand for 2-PE is rapidly increasing, which has attracted research focused on its production. Nevertheless, the use of organic reagents in the extraction process leads to the dilemma of high yield, poor quality 2-PE, as well as complex process redundancy. Therefore, a new safe and high-yield method for 2-PE production for food-grade application is provided here. In this study, we developed effective yeast strains for 2-PE production using both adaptive evolution and rational pathway engineering. Tolerance of the yeast strain to 2-PE was improved via an adaptive evolutionary method, expressing fusion proteins encoded by *ectyrB*-*llkdcA*-*scADH2*, as well as the utilization of L-glutamate oxidase, which was certified as an effective strategy to increase 2-PE production. Additionally, deleting *PDC5* provided a new way to increase 2-PE yield. The engineered yeast strain resulted in the considerable 2-PE titer of 4.02 g/L in a medium containing 6.7 g/L L-phenylalanine, yielding 0.8 mol/mol L-phenylalanine. This shows that the high yield strain was achieved by metabolic engineering in flasks without the ISPR technique, making it better than the 3.73 g/L observed in Wang’s research [11] and 0.62 mol/mol L-phenylalanine in Kim’s research [10]. This demonstrated that high and safe 2-PE production could be achieved by applying an efficient metabolic engineering strategy in higher 2-PE-tolerant strains. Additionally, with more fermentation processes applied, such as fed-batch fermentation methods with further condition optimization, significant improvement in natural 2-PE production would be achieved. This would be beneficial for the wide application of 2-PE in the food industry.

## Supporting information

S1 Fig Cell growth (lg y-axis scale) of strains JM00 and AD032 in the flask fermentation with 4 g/L phenylalanine (a) and 6.7 g/L phenylalanine (b) culture medium.
(TIFF)

S1 Table The primers used for enzyme expressing vector construction in this study. (DOCX)

S2 Table The primers used for fusion-protein expressing vector construction in this study. (DOCX)

S3 Table The primers used for gene knockout in this study. (DOCX)

S4 Table The primers used for transcription level analysis in this study. (DOCX)

S1 File Codon-optimized enzyme sequences in this study. (DOCX)
