# Supplementary figures and images for: Improvement of 2-phenylethanol production in Saccharomyces cerevisiae by evolutionary and rational metabolic engineering
Source: PLoS One. 2021 Oct 19;16(10):e0258180. doi: 10.1371/journal.pone.0258180 (PMC8525735; doi:10.1371/journal.pone.0258180)

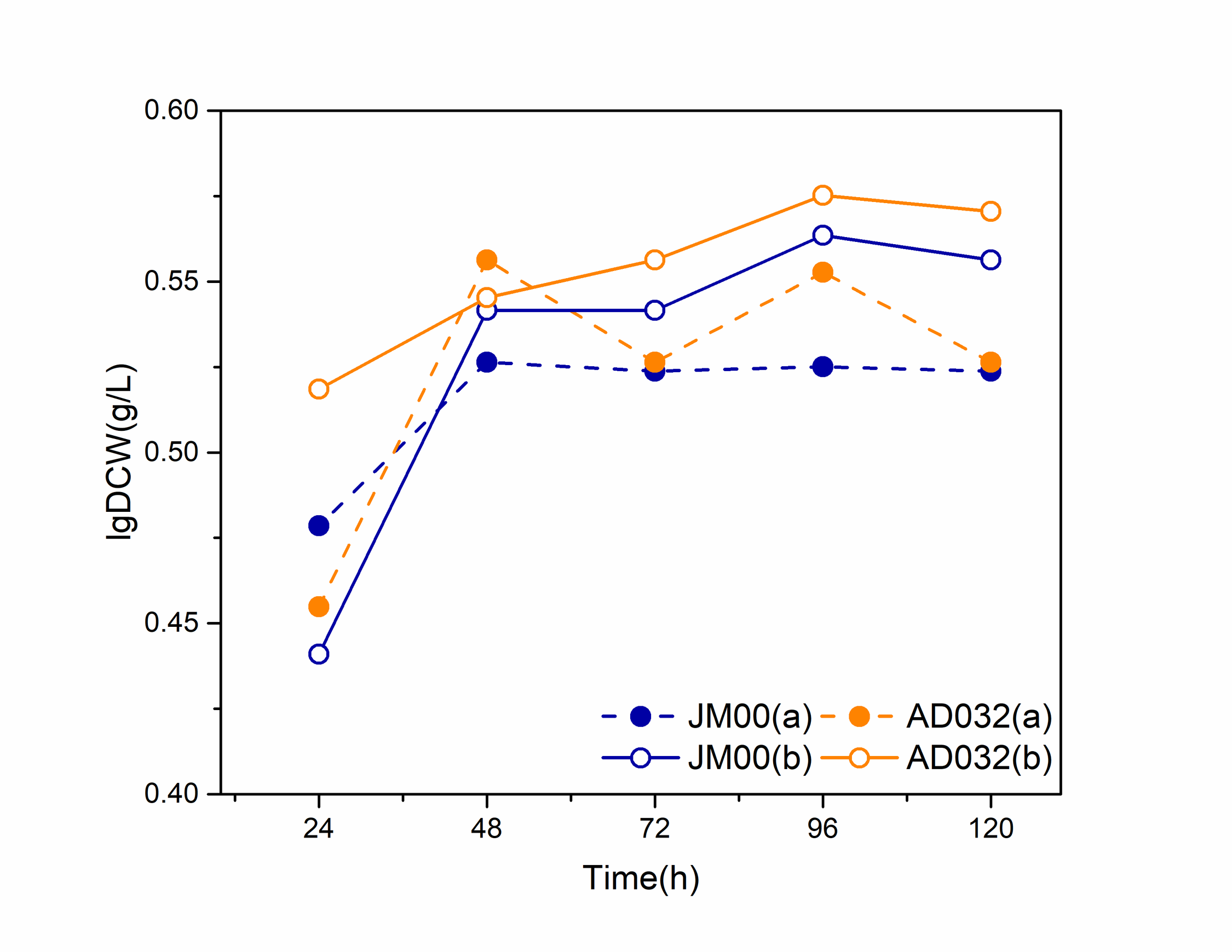

Supplement: S1 Fig — Cell growth (lg y-axis scale) of strains JM00 and AD032 in the flask fermentation with 4 g/L phenylalanine (a) and 6.7 g/L phenylalanine (b) culture medium. (TIFF) [file pone.0258180.s001.tiff]
