# Supplementary material for: Improvement of 2-phenylethanol production in Saccharomyces cerevisiae by evolutionary and rational metabolic engineering
Source: PLoS One. 2021 Oct 19;16(10):e0258180. doi: 10.1371/journal.pone.0258180 (PMC8525735; doi:10.1371/journal.pone.0258180)
Supplement: S1 Table — (DOCX) [file pone.0258180.s002.docx]

**Supporting information**

S1 Table The primers used for enzyme expressing vector construction in this study

| Gene | Template | Primer sequence (5’–3’) for amplification and ligation ^a^ |
| --- | --- | --- |
| *ARO8* | *S. cerevisiae* | Fwd: CGGGATCCCGATGACTTTACCTGAATCA(*Bam*HI)  Rev: ACGTCGACGCCTATTTGGAAATACC(*Sal*I) |
| *ARO9* | *S. cerevisiae* | Fwd: CGGGATCCCGatgactgctggttctgcccc(*Bam*HI)  Rev: CGGTCGACGCTCAACTTTTATAGTTGTCAAAA(*Sal*I) |
| *ARO10* | *S. cerevisiae* | Fwd: CGGGATCCCGTTAAGCATGGCACCTGTTA(*Bam*HI)  Rev: CGACGCGTCGCCTATTTTTTATTTCTTTTAA(*Mlu*I) |
| *ADH2* | *S. cerevisiae* | Fwd: CGGGATCCCGatgtctattccagaaactc(*Bam*HI)  Rev: ACGTCGACGCTTATTTAGAAGTGTCAACAACG(*Sal*I) |
| *tyrB* | *E. coli* | Fwd: cgggatcccgATGTTCCAGAAGGTCGAT(*Bam*HI)  Rev: ACGTCGACGC TTACATCACC GCAGCAAACG(*Sal*I) |
| *kdcA* | *Gene synthesis* | Fwd: CGGGATCCCGatgtatacagtaggagattacc(*Bam*HI)  Rev: ACGTCGACGCCTATTTATTTTGCTCAGCAA(*Sal*I) |
| *strlgox* | *Gene synthesis* | Fwd: GTCGAATTCATGACCACCGATACAGCACG(*Eco*RI)  Rev: CTAGGGATCCTTAGCTTGTTAAAGCCTCTTCACGC(*Bam*HI) |
| *kitlgox* | *Gene synthesis* | Fwd: GTCGAATTCATGGCCGAGACCGTTAT(*Eco*RI)  Rev: CTAGGGATCCTTAGCGTTGATGCACTTCCA(*Bam*HI) |

^a^ The underscores were the sites for restriction digest.
