# Supplementary material for: Improvement of 2-phenylethanol production in Saccharomyces cerevisiae by evolutionary and rational metabolic engineering
Source: PLoS One. 2021 Oct 19;16(10):e0258180. doi: 10.1371/journal.pone.0258180 (PMC8525735; doi:10.1371/journal.pone.0258180)
Supplement: S2 Table — (DOCX) [file pone.0258180.s003.docx]

**Supporting information**

S2 Table The primers used for fusion-protein expressing vector construction in this study

| Genes | Template | Primer sequence (5’–3’) for amplification ^a^ and ligation ^b^ |
| --- | --- | --- |
| *ARO8-kdcA* | *S. cerevisiae* | Fwd: CGGGATCCCGATGACTTTACCTGAATCA(*Bam*HI)  Rev: ctcctactgtatacat**ACTACCACCACCACC**TTTGGAAATACCAAATTCTTCG |
|  | *Gene synthesis* | Fwd: CGAAGAATTTGGTATTTCCAAA**GGTGGTGGTGGTAGT**atgtatacagtaggag  Rev: ACGTCGACGCCTATTTATTTTGCTCAGCAA(*Sal*I) |
| *ARO9-kdcA* | *S. cerevisiae* | Fwd: CGGGATCCCGatgactgctggttctgcccc(*Bam*HI)  Rev: ctcctactgtatacat**ACTACCACCACCACC**acttttatagttgtc |
|  | *Gene synthesis* | Fwd: gacaactataaaagt**GGTGGTGGTGGTAGT**atgtatacagtaggag  Rev: ACGTCGACGCCTATTTATTTTGCTCAGCAA(*Sal*I) |
| *tyrB-kdcA* | *E. coli* | Fwd: cgggatcccgATGTTCCAGAAGGTCGAT(*Bam*HI)  Rev: ctcctactgtatacat**ACTACCACCACCACC**CATCACCGCAGCAAACGCCT |
|  | *Gene synthesis* | Fwd: AGGCGTTTGCTGCGGTGATG**GGTGGTGGTGGTAGT**atgtatacagtaggag  Rev: ACGTCGACGCCTATTTATTTTGCTCAGCAA(*Sal*I) |
| *tyrB-kdcA-ADH2* | *E. coli* | Fwd: CGGGATCCCGatgtatacagtaggagattacc(*Bam*HI)  Rev: ctcctactgtatacat**ACTACCACCACCACC**CATCACCGCAGCAAACGCCT |
|  | *Gene synthesis* | Fwd: AGGCGTTTGCTGCGGTGATG**GGTGGTGGTGGTAGT**atgtatacagtaggag  Rev: gagtttctggaatagacat**ACTACCACCACCACC**tttattttgctcagcaaa |
|  | *S. cerevisiae* | Fwd: tttgctgagcaaaataaa**GGTGGTGGTGGTAGT**atgtctattccagaaactc  Rev: ACGTCGACGCTTATTTAGAAGTGTCAACAACG(*Sal*I) |
| *ARO8-ARO10-ADH2* | *S. cerevisiae* | Fwd: CGGGATCCCGATGACTTTACCTGAATCA(*Bam*HI)  Rev: caattgtaacaggtgccat**ACTACCACCACCACC**TTTGGAAATACCAAATTCTTCG |
|  | *S. cerevisiae* | Fwd: CGAAGAATTTGGTATTTCCAAA**GGTGGTGGTGGTAGT**ATGGCACCTGTTACAATTG  Rev: GAGTTTCTGGAATAGACAT**ACTACCACCACCACC**TTTTTTATTTCTTTTAAGTGCCGC |
|  | *S. cerevisiae* | Fwd: cagcggcacttaaaagaaataaaaaa**GGTGGTGGTGGTAGT**atgtctattccagaaac  Rev: ACGTCGACGCTTATTTAGAAGTGTCAACAACG(*Sal*I) |

^a^ The overstriking nucleotides were the linker between the enzymes.

^b^ The underscores were the sites for restriction digest.
