# Supplementary material for: Improvement of 2-phenylethanol production in Saccharomyces cerevisiae by evolutionary and rational metabolic engineering
Source: PLoS One. 2021 Oct 19;16(10):e0258180. doi: 10.1371/journal.pone.0258180 (PMC8525735; doi:10.1371/journal.pone.0258180)
Supplement: S3 Table — (DOCX) [file pone.0258180.s004.docx]

**Supporting information**

S3 Table The primers used for gene knockout in this study

| Target gene | Primer sequence (5’–3’) for knockout-cassette | Primer sequence (5’–3’) for whole gene |
| --- | --- | --- |
| *PDC1* | Fwd:gccagctaacttggtcgacttgaacgtcccagctaagttgttgcaaactccaattgacatgtctttgaagTCGAAAATTCTGCGTTCGG Rev:TTTCTTGAACAGTCAATTGCAAAGAACCGTCACCAATGAATAAGATAACTCTCTTCTTTGGATCAATTTCCGATCCTCTGGAGATGAGC | Fwd:gaaggtatgagatgggctgg  Rev:atgggacaagttgacccaag |
| *PDC5* | Fwd:CTTTCTTATTACACTTATTTCACATAATCAATCTCAAAGAGAACAACACAATACAATAACAAGAAGAACATCGAAAATTCTGCGTTCGG  Rev:CCTAAACATCTATAACCTTCAAAAGTAAAAAAATACACAAACGTTGAATCATGAGTTTTATGTTAATTAGCGATCCTCTGGAGATGAGC | Fwd:CAACCAGGTGAGAATCCTTC  Rev:TAATCTTTAACCTGGAAGA |
| *PDC6* | Fwd:TTATGTATTCAAACTGTGTAAATTTATTTATTTGCAACAATAATTCGTTTTTGAGTACACTACTAATGGCTCGAAAATTCTGCGTTCGG  Rev:TTTACCAATATGTATAAAAGGCGGCTGTTTGAAGCCATTCTATCTTAATCTTGTGCTATTGCAGTCCTCTCGATCCTCTGGAGATGAGC | Fwd:AAAGAGATGAGCCAAAGCA  Rev:TAGTGTAGTAGTGATAAACT |
| *ARO10* | Fwd:GATACTCAAAACAAGTTGACGCGACTTCTGTAAAGTTTATTTACAAGATAACAAAGAAACTCCCTTAAGCTCGAAAATTCTGCGTTCGG  Rev:AGGGTTTTTTATGTGTTAATGAACAGAAAACGAACAATTGGTAGCAGTGTTTTATAATTGCGCCCACAAGCGATCCTCTGGAGATGAGCCGATCCTCTGGAGATGAGC | Fwd:TTTATTGTGTTGAGTAAAGT  Rev:ACGTCTCCCCGCCCCTGCCT |
| *THI3* | Fwd:cataactactaaaacgcaccgtcgtcattctgaagatgaattctagctatacacagagatatgcactgccTCGAAAATTCTGCGTTCGG  Rev:tcgcattcgagcggtaatcatgagggtccctggtagtagggcggagagatcagatcagtatccaacttgaCGATCCTCTGGAGATGAGC | Fwd:TAAACGAAACAGACCCGC  Rev:TGGAAGTGGAAAGTTGACG |
| *ARO8* | Fwd:cggaattcgcATGACTTTACCTGAATCAAAAGACTTTTCTTACTTGTTTTCGGATGAAACCAATGCTCGTTCGAAAATTCTGCGTTCGG  Rev:ccaagcttggCTAgtggtggtggtggtggtgTTTGGAAATACCAAATTCTTCGTATAAAGTATCACCTAACGATCCTCTGGAGATGAGC | Fwd:CCGatgactttacctgaatc  Rev:GCCGGCTATTTGGAAATACC |
| *ARO9* | Fwd:atgactgctggttctgccccccctgttgattacacttccttaaagaagaacttccaaccgtttctctccaTCGAAAATTCTGCGTTCGG  Rev:caacttttatagttgtcaaaaaattcttttatgccactaccgattcttttggaagcttcaatcaactgatCGATCCTCTGGAGATGAGC | Fwd:atgactgctggttctgcccc  Rev:tttgacaactataaaagttg |
