# Supplementary material for: Improvement of 2-phenylethanol production in Saccharomyces cerevisiae by evolutionary and rational metabolic engineering
Source: PLoS One. 2021 Oct 19;16(10):e0258180. doi: 10.1371/journal.pone.0258180 (PMC8525735; doi:10.1371/journal.pone.0258180)
Supplement: S4 Table — (DOCX) [file pone.0258180.s005.docx]

**Supporting information**

S4 Table The primers used for transcription level analysis in this study

| *Gene* | Primer sequence (5’–3’) for transcription level analysis |
| --- | --- |
| *ACT1* | Fwd: CTGGTTTCTCTCTACCTCACGC  Rev: TCTCTTTCAGCAGTGGTGGAG |
| *PDC1* | Fwd: TTGAAGGTATGAGATGGGC  Rev: CTTGAGCAGAGATGGATGG |
| *PDC5* | Fwd: CAAGTCAACTGTAACACCGTCTTC  Rev: CGTAACCATCAGCAGCATAGG |
| *PDC6* | Fwd: TGTTATCATTTCCGAGACCG  Rev: GCAAAGGCAGCACCTAAAG |
| *ARO10* | Fwd: GCAAGACCACTCAAACGCTC  Rev: TGGATTGTCATCTGTGCTGC |
| *THI3* | Fwd: ACAACCTTTGGTGTAGGCG  Rev: CCATTGCCCAGAGTATGATG |
| *ARO8* | Fwd: AAAGTTCTCCAAAGCAGGAC  Rev: ACCAGTAATCCAACCCAATC |
| *ARO9* | Fwd: GGGTCCAACGATTCAATG  Rev: CGAAGGTCAGGTTCATCTTG |
| *ADH2* | Fwd: ATTGTTTACCTCGCTCGG  Rev: ATTGATGATACCGTGGGC |
| *tyrB* | Fwd: CGCTTCGCTTTATTTACCG  Rev: CCAAGGGTTTGAATGGTTG |
| *KdcA* | Fwd: GCTGTTGATGAATCTTTGCC  Rev: ACCTGTTGAGGAGTCCGTAAG |
