# Supplementary material for: Improvement of 2-phenylethanol production in Saccharomyces cerevisiae by evolutionary and rational metabolic engineering
Source: PLoS One. 2021 Oct 19;16(10):e0258180. doi: 10.1371/journal.pone.0258180 (PMC8525735; doi:10.1371/journal.pone.0258180)
Supplement: S1 File — (DOCX) [file pone.0258180.s006.docx]

S1 File. Codon-optimized enzyme sequences in this study.

KdcA 1644 bp

ATGTATACAGTAGGAGATTACCTGTTAGACCGATTACACGAGTTGGGAATTGAAGAAATTTTTGGAGTTCCTGGTGACTATAACTTACAATTTTTAGATCAAATTATTTCACGCGAAGATATGAAATGGATTGGAAATGCTAATGAATTAAATGCTTCTTATATGGCTGATGGTTATGCTCGTACTAAAAAAGCTGCCGCATTTCTCACCACATTTGGAGTCGGCGAATTGAGTGCGATCAATGGACTGGCAGGAAGTTATGCCGAAAATTTACCAGTAGTAGAAATTGTTGGTTCACCAACTTCAAAAGTACAAAATGACGGAAAATTTGTCCATCATACACTAGCAGATGGTGATTTTAAACACTTTATGAAGATGCATGAACCTGTTACAGCAGCGCGGACTTTACTGACAGCAGAAAATGCCACATATGAAATTGACCGAGTACTTTCTCAATTACTAAAAGAAAGAAAACCAGTCTATATTAACTTACCAGTCGATGTTGCTGCAGCAAAAGCAGAGAAGCCTGCATTATCTTTAGAAAAAGAAAGCTCTACAACAAATACAACTGAACAAGTGATTTTGAGTAAGATTGAAGAAAGTTTGAAAAATGCCCAAAAACCAGTAGTGATTGCAGGACACGAAGTAATTAGTTTTGGTTTAGAAAAAACGGTAACTCAGTTTGTTTCAGAAACAAAACTACCGATTACGACACTAAATTTTGGTAAAAGTGCTGTTGATGAATCTTTGCCCTCATTTTTAGGAATATATAACGGGAAACTTTCAGAAATCAGTCTTAAAAATTTTGTGGAGTCCGCAGACTTTATCCTAATGCTTGGAGTGAAGCTTACGGACTCCTCAACAGGTGCATTCACACATCATTTAGATGAAAATAAAATGATTTCACTAAACATAGATGAAGGAATAATTTTCAATAAAGTGGTAGAAGATTTTGATTTTAGAGCAGTGGTTTCTTCTTTATCAGAATTAAAAGGAATAGAATATGAAGGACAATATATTGATAAGCAATATGAAGAATTTATTCCATCAAGTGCTCCCTTATCACAAGACCGTCTATGGCAGGCAGTTGAAAGTTTGACTCAAAGCAATGAAACAATCGTTGCTGAACAAGGAACCTCATTTTTTGGAGCTTCAACAATTTTCTTAAAATCAAATAGTCGTTTTATTGGACAACCTTTATGGGGTTCTATTGGATATACTTTTCCAGCGGCTTTAGGAAGCCAAATTGCGGATAAAGAGAGCAGACACCTTTTATTTATTGGTGATGGTTCACTTCAACTTACCGTACAAGAATTAGGACTATCAATCAGAGAAAAACTCAATCCAATTTGTTTTATCATAAATAATGATGGTTATACAGTTGAAAGAGAAATCCACGGACCTACTCAAAGTTATAACGACATTCCAATGTGGAATTACTCGAAATTACCAGAAACATTTGGAGCAACAGAAGATCGTGTAGTATCAAAAATTGTTAGAACAGAGAATGAATTTGTGTCTGTCATGAAAGAAGCCCAAGCAGATGTCAATAGAATGTATTGGATAGAACTAGTTTTGGAAAAAGAAGATGCGCCAAAATTACTGAAAAAAATGGGTAAATTATTTGCTGAGCAAAATAAATAG

LGOXstr 2106 bp

ATGACCACCGATACAGCACGCCGTCACACCGGTGCAGAGCGCGCCAATGAAATGACCTATGAACAGCTGGCCCGCGAATTACTGCTGGTTGGCCCGGCACCTACCAATGAGGACCTGAAACTGCGCTACCTGGATGTGCTGATCGACAACGGTCTGAATCCGCCTGGCCCGCCGAAGCGCATTCTGATCGTTGGTGCAGGCATTGCCGGTCTGGTGGCCGGCGATTTACTGACCCGTGCAGGCCACGATGTGACCATTCTGGAAGCAAATGCCAATCGTGTGGGCGGCCGTATTAAGACCTTCCATGCTAAAAAAGGCGAACCGAGCCCTTTCGCCGATCCGGCACAGTATGCCGAAGCCGGCGCAATGCGTCTGCCGAGCTTTCACCCGCTGACCCTGGCACTGATCGATAAGCTGGGCCTGAAGCGTCGTTTATTTTTTAACGTGGATATCGACCCGCAAACCGGTAATCAGGATGCCCCGGTGCCGCCTGTTTTTTACAAGTCTTTTAAAGATGGTAAAACCTGGACCAACGGCGCACCTAGTCCGGAGTTCAAAGAGCCGGATAAGCGTAACCACACCTGGATTCGCACCAACCGCGAACAGGTTCGCCGCGCACAATATGCAACCGACCCGAGCAGTATCAATGAAGGCTTTCACCTGACCGGTTGCGAAACCCGCCTGACCGTGAGCGATATGGTGAATCAAGCCCTGGAGCCGGTTCGTGACTATTACAGCGTGAAGCAGGACGATGGCACCCGTGTTAACAAACCGTTTAAAGAATGGTTAGCCGGCTGGGCCGATGTGGTGCGCGATTTTGATGGCTACAGCATGGGTCGTTTCCTGCGCGAGTATGCCGAGTTTAGCGATGAGGCAGTGGAAGCAATCGGCACCATCGAGAATATGACCAGCCGCCTGCATCTGGCCTTCTTCCACAGCTTTCTGGGCCGTAGCGACATTGATCCGCGTGCCACATACTGGGAGATCGAAGGTGGTAGCCGCATGTTACCGGAAACCCTGGCAAAGGATCTGCGCGACCAAATCGTTATGGGCCAGCGCATGGTGCGCCTGGAATATTATGATCCGGGTCGCGATGGTCATCACGGTGAATTAACCGGTCCGGGTGGCCCGGCAGTTGCCATTCAGACCGTTCCGGAAGGCGAACCGTATGCCGCCACCCAAACCTGGACCGGCGATCTGGCAATTGTGACCATTCCGTTCAGCAGCCTGCGCTTTGTTAAGGTGACCCCGCCGTTTAGCTACAAGAAACGCCGCGCCGTGATCGAGACACATTACGATCAGGCAACCAAAGTGTTACTGGAATTTAgCCgCCGCTGGTGGGAGTTTACCGAGGCCGATTGGAAGCGTGAACTGGATGCCATTGCACCGGGTCTGTACGATTACTATCAGCAGTGGGGCGAGGATGACGCCGAGGCAGCCTTAGCACTGCCTCAGAGCGTGCGCAACTTACCGACCGGTCTGTTAGGCGCACATCCGAGTGTTGACGAAAGTCGCATCGGCGAAGAGCAGGTGGAATATTATCGCAACAGCGAACTGCGCGGTGGTGTGCGTCCGGCAACCAACGCTTATGGTGGCGGCAGCACCACCGACAATCCGAATCGCTTCATGTACTACCCGAGCCATCCGGTGCCGGGCACACAAGGTGGTGTGGTGCTGGCAGCCTATAGCTGGAGCGACGATGCAGCACGCTGGGACAGCTTTGACGACGCCGAACGCTACGGTTACGCCCTGGAAAATCTGCAAAGCGTGCATGGTCGCCGCATCGAAGTTTTCTATACAGGCGCAGGCCAGACCCAGAGCTGGTTACGTGACCCGTATGCATGTGGTGAGGCCGCAGTGTATACCCCGCACCAGATGACCGCATTTCACCTGGACGTTGTGCGTCCGGAAGGTCCTGTGTATTTTGCCGGTGAGCATGTGAGCCTGAAACATGCTTGGATTGAAGGCGCAGTGGAAACCGCAGTTCGTGCCGCCATCGCCGTGAATGAAGCACCGGTTGGTGATACAGGCGTTACCGCCGCAGCCGGTCGCCGCGGTGCAGCAGCAGCCACCGAGCCGATGCGTGAAGAGGCTTTAACAAGCTAA

LGOXkit 1764 bp

ATGGCCGAGACCGTTATCGGTCGTCGTGCCCTGCTGACCACAGCAGCCGCAGCAGGTGCCTTAGCCGCAGGTGCAACCACCTTAGCCTGTGGTCCGCCGAGCGCACGCACAGATTTCGCACGCAGCATGGATCGTAGTGTGGAACTGGCACGTGAAATGCTGGGTGTGGATGCCGCCGGTAACGATCTGCGTTTAACCTATCTGCGCACCCTGATCGACACCGGCTTACCGGGCACAGCAGCACCGAAACGCGTTCTGGTTATCGGCGCCGGTCCCGCTGGTCTGACCGCAGCCAATCTGCTGGCAGATGCCGGTCATCGCGTGACCGTTATTGAAGCCAATGGCAGCCGTACAGGCGGCCGTGTTAAAACCTTCCGCGGTATGTTCAGTGACCCGAATCTGTATGCCGAGGCAGGTGCAATGCGCCTGCCGAGTGCACATCCGATGGTTCTGGCCCTGGCCGACAAACTGGGTCTGCGTCGCCGCCAGTTTCATAACGCAGATGTGGCACCGGAAGCACGCAGCGCCGCCGTTACACCTGTGGTGTATCGCAGCTTTACCGGCGAACAGTGGAGCAACGGTCCGGCAGCAGAATTTCGTCCGCCGCCTGCCGCCGGCCGTACCCTGATTAACACCAACGGCCGCATCGTGACACGTGCCGAGTATGCAGGTGATCCGGCCGCCCTGCATCGCGATTTCGGCGTTGATTTAACCGCACCGGCCCGTGTTGCCTTAGATAGCGCCCTGCATAAAGTGGGCGTTCCTGATGACTGGCCGATCGAACGCCGCATTGATGGCTGGGCCAAGGTTTTCAACACCTATGAGGACTATAGCACCCACCGTTATCTGATGGAACATGGCTGGAGTCTGGCACACCTGGCAACCGTGGGCACCCTGGAAAACCTGACCAGCCGCCTGCATTACGGCGTTATTAGCGCTATGGTTGATCACGCCCTGATTCGCCCGGACGCAAGTTACTGGGAACTGGAGGGTGGCACCGCAACCCTGACAGATGCACTGACCCGTAAGCTGGCCCCGGCCGTTCGTCAAGGTCGCCGTATGACCCATCTGGAGCAGACAGATCGCGGCGTTAAGGTGTGGACCACAGCCGAAAGTGGCAGCGAGCATACCGATGGCGCCCCTATGGACCCTATCGAGACCTTTGAGGGTGATTACGCCATCCTGGCCATCCCTCTGACCGCCACCCGCTTTTGCACCTTTGATCCGCCGCTGAGCTATCCGACACGCCGCGCAATTACCGAACTGCATCACGACGCCGCAACCAAGGTGCTGCTGGAGTTTAAGACACGCTTCTGGGAACAGGGCGTTCGTGGTTTCCGTGGCGGTGGTTGTGTTAGTGACTGCCCGAACCGTTTCACCTACTTTCCGAGCCATGTGCCTGAAAGCGATGGCGGTGTGGTTCTGGCCAGCTATACCTGGAGCGATGATGCCATGCGTTGGGATAGTCTGACCGAGAGTGAGCGTGTTCACTTCGCCCTGGCCGGTATGCGCCGTATGTTTGGCCCGCGCGTTGACACCGAGTTCACCGGCGTTGGTGTGAGCCAGAGCTGGCAGCGTGCCCGTTACGCCTTAGGTGAAGCCGTTATCCCGACTCCTGGTCAGCTGCATGAGCATCATGCCGCCACCCGTACAATCGAAGGCCGTATTCATCTGGCCGGCGATCATACCACCCTGAAACCGGCCTGGATTGAAGGTGCATTAGAAAGCGCCGTGCGTACAGCCCTGGAAGTGCATCAACGCTAA
